# Sphingosine Kinases are Involved in Macrophage NLRP3 Inflammasome Transcriptional Induction

**DOI:** 10.3390/ijms21134733

**Published:** 2020-07-02

**Authors:** Shahzad Nawaz Syed, Andreas Weigert, Bernhard Brüne

**Affiliations:** 1 Institute of Biochemistry I, Faculty of Medicine, Goethe-University Frankfurt, 60590 Frankfurt, Germany; syed@biochem.uni-frankfurt.de (S.N.S.); weigert@biochem.uni-frankfurt.de (A.W.); 2 Project Group Translational Medicine and Pharmacology TMP, Fraunhofer Institute for Molecular Biology and Applied Ecology, 60596 Frankfurt, Germany; 3 German Cancer Consortium (DKTK), Partner Site Frankfurt, 60590 Frankfurt, Germany; 4 Frankfurt Cancer Institute, Goethe-University Frankfurt, 60596 Frankfurt, Germany

**Keywords:** Macrophage, sphingosine-1-phosphate, NLRP3 inflammasomes, inflammation, IL-1β, renal cell carcinoma, psoriasis

## Abstract

Recent studies suggested an important contribution of sphingosine-1-phospate (S1P) signaling via its specific receptors (S1PRs) in the production of pro-inflammatory mediators such as Interleukin (IL)-1β in cancer and inflammation. In an inflammation-driven cancer setting, we previously reported that myeloid S1PR1 signaling induces IL-1β production by enhancing NLRP3 (NOD-, LRR- and Pyrin Domain-Containing Protein 3) inflammasome activity. However, the autocrine role of S1P and enzymes acting on the S1P rheostat in myeloid cells are unknown. Using human and mouse macrophages with pharmacological or genetic intervention we explored the relative contribution of sphingosine kinases (SPHKs) in NLRP3 inflammasome activity regulation. We noticed redundancy in SPHK1 and SPHK2 activities towards macrophage NLRP3 inflammasome transcriptional induction and IL-1β secretion. However, pharmacological blockade of both kinases in unison completely abrogated NLRP3 inflammasome induction and IL-1β secretion. Interestingly, human and mouse macrophages demonstrate varied responses towards SPHKs inhibition and IL-1β secretion. Clinical datasets of renal cell carcinoma and psoriasis patients showed a positive correlation between enzymes affecting the S1P rheostat with NLRP3 inflammasome components expression, which corroborates our finding. Our data provide a better understanding on the role of SPHKs and de novo synthesized S1P in macrophage NLRP3 inflammasome activation.

## 1. Introduction

Macrophages are dynamic and versatile players of sterile and smoldering inflammation, due to the fact that they can mount, control, as well terminate inflammation [1,2]. In an inflammatory setting, including cancer, macrophages can be primed and activated by a variety of damage-associated molecular patterns (DAMPs), including toll-like receptor (TLR) agonists such as HMGB1 or BD-2. These agonists are produced by injured or damaged cells, which provokes the secretion of interleukin-1 beta (IL-1β) and IL-18, amongst others. The secretion of IL-1β from primed macrophages depends on the formation of a large molecular scaffold that contains cytosolic pattern recognition receptors, adaptor proteins, and caspase-1, altogether composing the inflammasome. The pattern recognition receptor NOD-like receptor pyrin domain containing 3 (NLRP3) is well characterized, most relevant to sterile inflammatory responses, and has been implicated in sensing non–microbial-originated DAMPs [3,4,5,6]. Any trigger that activates the transcription factor nuclear factor kappa B (NF-κB), such as ligands for IL-1R1, TLRs, NOD-like receptors (NLRs), and the cytokine receptors for tumor necrosis factor (TNFR) are considered as the priming stimuli, also known as “signal 1,” for NLRP3 inflammasome activation [7,8]. Signal 1 is required for upregulating the transcription of both pro-IL-1β and NLRP3 since pro-IL-1β is not constitutively expressed and basal expression of NLRP3 is inadequate for efficient inflammasome formation [9]. The “signal 2” for inflammasome activation derives from a diverse group of agonists that trigger the specific activation of NLRP3, assembly of the inflammasome complex that requires its interaction with the pyrin domain of ASC (apoptosis-associated speck-like protein containing carboxy-terminal CARD; encoded by *PYCARD*), and finally culminates in the activation of caspase-1 [7,10]. Caspase-1 then cleaves pro-IL-1β into its active form as a prerequisite of its release and activity at the IL-1 receptor. IL-1β is a key inflammatory cytokine associated with many pathologies and is responsible for triggering multiple downstream inflammatory pathways [11]. We recently demonstrated that in tumor-associated macrophages NLRP3 inflammasome activation and subsequent IL-1β release is downstream of sphingosine-1-phosphate receptor 1 (S1PR1) signaling [12]. S1PR1 was also involved in promoting ATP release, at least in a murine macrophage cell line, which is one of the triggers of NLRP3 inflammasome activation [13]. Furthermore, the release of cathepsin B from lysosomes was associated with S1PR2 signaling [14] and the levels of IL-1β and IL-18 were reduced in serum of S1PR2-deficient mice challenged with endotoxin [15]. Thus, S1P via S1PR1/2 may cooperate toward NLRP3 inflammasome assembly and activation, promoting IL-1β maturation. However, if the inflammasome is also regulated at the level of S1P kinases is unknown.

Sphingosine-1-phosphate (S1P) is an important membrane derived, pleotropic signaling lipid that plays prominent roles in several diseases [16,17,18,19]. Especially in the context of inflammation, this lipid been shown to regulate various cellular events such as chemotaxis, apoptosis, phagocytosis, and macrophage polarization [19,20]. Intracellularly, S1P levels are dynamically regulated at picomolar concentrations by its synthesis and degradation by various enzymes, including sphingosine kinase (SPHKs), S1P lyases (SGPLs), and S1P phosphatases (SGPPs). S1P is generated from sphingosine by phosphorylation, which is carried out by two metabolically redundant kinases, i.e., SPHK1 and SPHK2. S1P binds to its specific G-protein-coupled receptors (S1PR1–S1PR5) to elicit cell responses. Besides, it can also bind to intracellular targets such as histone deacetylases and TNFR-associated factor 2 (TRAF2) [21,22]. Intracellular S1P can also act as a second messenger to trigger calcium release from the endoplasmic reticulum [23,24,25]. In spite of demarcated receptor-dependent and independent actions of S1P, the autocrine and paracrine functions of S1P in innate immune cells such as macrophages are still elusive, especially in the context of NLRP3 inflammasome activation. Furthermore, factors upstream to S1PRs were not systematically investigated as a putative therapeutic targets in inflammation and cancer. In this study, we sought to characterize autocrine and paracrine functions of S1P in macrophages and to define components of S1P production or its signaling machinery that is best suited to target S1P-dependent NLRP3 inflammasome activation and IL-1β production.

## 2. Results

### 2.1. Sphingosine Kinases are Involved in Signal 1 of NLRP3 Inflammasome Activation

**Figure 1:**
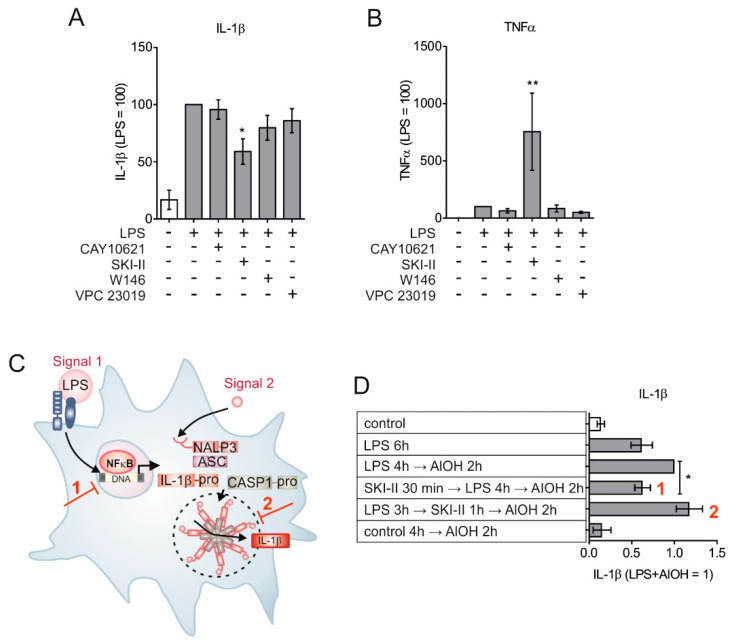
Sphingosine kinases are involved in “signal 1” of the NLRP3 inflammasome. Primary human macrophages were preincubated for 30 min with 5 µM CAY10621, 10 µM SKI-II, and 1 µM each of W146 and VPC 23019. Cells were then stimulated with 10 ng/mL LPS with or without inhibitors for 2 h. Agonists and inhibitors were washed off and cells were further cultivated in fresh media for another 24 h. Cell free supernatants were analyzed for (**A**) IL-1β and (**B**) TNF-α by cytometric bead array (CBA). Data are means ± SEM, *n* = 6 donors. (**C**) Schematic depiction of two signals for NLRP3 inflammasome activation. Red numbers indicate intervention with signal 1 and signal 2 respectively. (**D**) Macrophages were left untreated, treated with 100 ng/mL LPS, 1 µg/mL AlOH and 10 µM SKI-II for 1–6 h in a permutation and combination depicted in the table and IL-1β levels were measured in cell-free supernatant using CBA. Red numbers indicated interventions as depicted in **C**. Data are means ± SEM, *n* ≥ 10 donors. * *p* < 0.05; ** *p* < 0.001; *p* values were calculated using one-sample *t* test (**A**, **C**, **D**).

**Figure 2:**
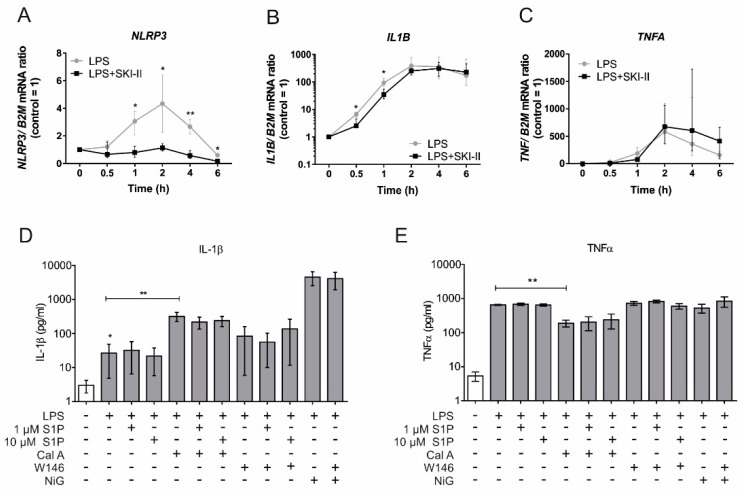
S1P kinases affect transcription of inflammasome components, without showing paracrine effects. (**A**–**C**) Primary human macrophages were pre-incubated with 10 µM SKI-II for 30 min then stimulated with 100 ng/mL LPS for up to 6 h. Samples were harvested at different time points as presented on X-axis and gene expression was analyzed by qPCR of (**A**) NLRP3, (**B**) IL-1β and (**C**) TNF-α. Data are means ± SEM, *n* ≥ 5 donors for each time points. (**D**, **E**) Cells were stimulated for 4 h with 500 ng/mL LPS. After washing LPS, cells were treated for 20 min with 25 nM calyculin A or 1 µM W146. Cells were again washed and treated for 2 h with 25 nM calyculin A, 1 µM W146, 1 and 10 µM S1P. Afterward, 6.7 µM nigericin was added for final 30 min. Cell-free supernatant containing (**D**) IL-1β and (**E**) TNF-α in last 2 h of treatments were measured by CBA. Data are means ± SEM, *n* ≥ 6 donors. * *p* < 0.05; ** *p* < 0.001; *p* values were calculated using two-tailed Student’s *t*-test.

To investigate the relative contribution of SPHKs in autocrine and paracrine S1PR activation, subsequent NLRP3 inflammasome activation, and IL-1β maturation, we activated the NLRP3 inflammasome in primary human macrophages by priming them with lipopolysaccharides (LPS) in combination with the inflammasome assembler aluminum hydroxide (AlOH). NLRP3 activation was carried out in the presence of the SPHK1 selective inhibitor (CAY10621) [26], the SPHK1/ SPHK2 inhibitor (SKI-II) [27,28,29], an S1PR1 antagonist (W146) [12] and the S1PR1/S1PR3 antagonist (VPC 23019) [30]. Mature IL-1β, released in the supernatant, was measured by the cytometric bead array (CBA), with a capture antibody that detects only mature IL-1β, as a function of NLRP3 inflammasome activation (Figure 1A). SKI-II, which blocks both SPHKs, significantly reduced IL-1β levels in the cell supernatant. All other treatments, including the selective inhibition of SPHK1, failed to alter IL-1β levels. Interestingly, blocking both S1P kinases by SKI-II provoked a massive release of TNF-α (Figure 1B). Reduced levels of mature IL-1β could be ascribed to either reduced expression, reduced inflammasome activity, or both, since a fully active NLRP3 inflammasome requires “signal 1” to induce transcription and “signal 2” for inflammasome assembly [31] (Figure 1C). To decipher at which stage SPHKs interfere with NLRP3 inflammasome activation primary human macrophages were sequentially incubated for 30 min with SKI-II, followed by 4 h with LPS. Afterwards inflammasome assembly was triggered for 2 h with AlOH (Figure 1D). Blocking sphingosine kinases prior to “signal 1” significantly reduced the release of mature IL-1β, whereas interfering with S1P kinases after LPS stimulation and prior to or simultaneously with inflammasome assembly had no influence on IL-1β release (Figure 1D). Conclusively, sphingosine kinase activities may affect transcriptional responses of the inflammasome in macrophages. To test this hypothesis, we measured expression kinetics of NLRP3 inflammasome components in the presence of SKI-II. As shown in Figure 2A, *NLRP3* expression was significantly reduced when macrophages were treated with SKI-II and expression of *IL1B* was attenuated as well (Figure 2B). However, SKI-II treatment had no influence on *TNFA* expression (Figure 2C). These results imply that indeed SPHKs activity can be linked to the transcription of NRLP3 inflammasome component.

The above findings so far did not fully rule out the involvement of S1P as a signal 2 for NLRP3 inflammasome activation and there are reports that at least sphingosine can fulfill this role [32,33]. To further investigate a potential role of a paracrine S1P action on the NLRP3 inflammasome activation, primary human macrophages were treated for inflammasome activation (LPS and AlOH), while S1P was exogenously provided. IL-β secretion was not potentiated with exogenous S1P even at a 10 µM concentration, which surpasses the concentration required to activate S1P receptors (Figure 2D). Furthermore, FTY720 a structural analogue of S1P that gets phosphorylated by sphingosine kinases to resemble S1P, is known to increase the activity of phosphatase PP2A, which is being implicated in inflammasome activation [32,34]. However, unlike mouse macrophages [32], human macrophages did not show any response to calyculin A, a serine/threonine phosphatase (PP1/PP2A) inhibitor, which failed to reduce IL-1β secretion. On the contrary, PP1/PP2A inhibition enhanced IL-1β release, with no impact of exogenous S1P supplementation (Figure 2D). We also ruled the role of exogenous S1P in NLRP3 inflammasome activation out, by probing S1P signaling in potassium efflux, which emerged as a common denominator in the both canonical [35,36] and non-canonical [37,38] NLRP3 inflammasome activation. Treatment with the potassium ionophore nigericin (6.7 µM) for 30 min after LPS stimulation provoked a massive release of mature IL-1β, which was unaffected by blocking autocrine signaling of S1P using 1 µM of the S1PR1 antagonist W146 (Figure 2D). ATP-mediated NLRP3 inflammasome activation [4] was also unaffected by exogenous S1P or S1PR1 blockage (data not shown). As expected, PP1/PP2A inhibition revealed anti-inflammatory effects in macrophages as TNF-α secretion upon LPS-stimulation was reduced (Figure 2E). At the same time, data in Figure 2E suggest that the compounds used in Figure 2D have no major side effects in terms of their inflammatory potential in human macrophages as the levels of TNFα with these treatments were comparable.

Together, these data indicate that the activities of both, SPHK1 and SPHK2 are affecting the expression of NLRP3 inflammasome components without auto/ or paracrine activation of S1PRs.

### 2.2. Sphk1 and Sphk2 Redundancy for Mouse Macrophage Nlpr3 Inflammasome Expression

**Figure 3:**
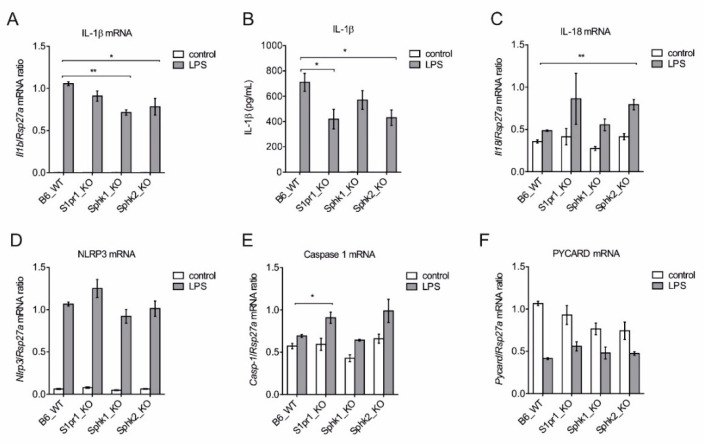
Relative contribution of *Sphk1*, *Sphk2,* and *S1pr1* in the expression of individual NLRP3 inflammasome components. BMDMs from B6 WT, *S1pr1*-LysM-Cre mice, *Sphk1* KO, and *Sphk2* KO mice were stimulated with 100 ng/mL LPS for 3 h before mRNA expression analysis by qPCR.. mRNA expression of (**A**) *Il1b*, (**B**) determination of mature IL-1β release by CBA after 7 h, (**C**) *Il18*, (**D**) *Nlpr3*, (**E**) *Casp1* and (**F**) *Pycard* expression was analyzed. Data are means ± SEM, *n* ≥ 4 mice. * *p* < 0.05; ** *p* < 0.001; *p* values were calculated using two-tailed Student’s *t*-test.

We then tried to delineate the relevance of individual S1P kinases by using bone marrow-derived macrophages (BMDM) from B6 wild type mice (WT) and mice deficient in *Sphk1*, *Sphk2* and *S1pr1,* for analyzing the expression of NLRP3 inflammasome components. LPS strongly induced *Il1b* expression in BMDMs of all mouse strains, however there was a significant reduction in *Il1b* expression in *Sphk1* and *Sphk2* deficient cells (Figure 3A). When the release of mature IL-1β from these cells was measured by CBA, *S1pr1* and *SphK2* deficient cells showed a lower IL-1β release (Figure 3B). The cytokine IL-18 also depends on NLRP3 inflammasome activity for maturation but showed week expression compared to *Il1b.* However, *Sphk2* deficiency potentiated its expression upon LPS stimulation (Figure 3C). Expression of the inflammasome component *Nlrp3* was drastically enhanced without significant differences among individual mouse strains (Figure 3D), whereas expression of caspase-1 (*Casp1*) was significantly upregulated only in *S1pr1* deficient macrophages (Figure 3E). *Pycard* (also known as ASC), a subunit of inflammasome, was downregulated upon LPS stimulation in cells of all strains (Figure 3D).

These data suggest that SPHKs play a role in the transcription of *Il1b*, whereas S1PR1 signaling might play a role in NLRP3 inflammasome activation.

### 2.3. Role of S1PR1 in NLRP3 Inflammasome Activation and Cytokines/Chemokine Production

**Figure 4:**
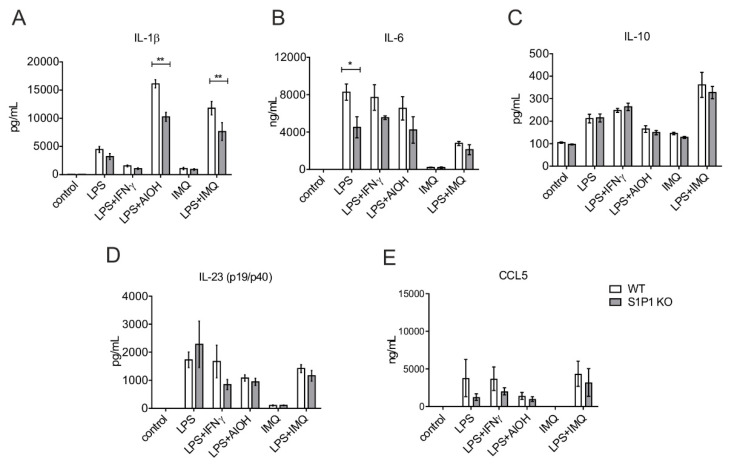
Role of S1PR1 in NLRP3 inflammasome activation. WT and S1PR1 KO macrophages (BMDMs) were treated with 100 ng/mL of LPS and IFNγ each, 10 µg/mL IMQ and 100 µg/mL AlOH for 16 h. Cell-free supernatants were harvested and analyzed by CBA for (**A**) IL-1β, (**B**) IL-6, (**C**) IL-10, (**D**) IL-23 (p19/p40) and (**E**) CCL5. Data are means ± SEM, *n* = 4 mice. * *p* < 0.05; ** *p* < 0.001; *p* values were calculated using two-way ANOVA with Sidak correction.

Although exogenous S1P was not potent enough to trigger the NLRP3 inflammasome in primary human macrophages upon LPS stimulation in the absence of signal 2 (Figure 2D), mouse macrophages have been shown to respond differently to these stimuli [39,40]. Still, the role of S1PR1 as a signal 2 during NLRP3 inflammasome activation is unknown. We explored the relevance of S1PR1 in NLRP3 inflammasome activation using BMDMs from myeloid-specific S1PR1-ablated mice. B6 WT and S1PR1 KO macrophages were treated with LPS alone or in combination with IFNγ, AlOH, and imiquimod (IMQ). The release of cytokines and CCL5 was measured in the cell-free supernatant by CBA. LPS induced the secretion of mature IL-1β, whereas IMQ produced only an insignificant release. However, NLRP3 signal 2 i.e., AlOH provoked much higher IL-1β secretion over LPS alone. S1PR1 deficient macrophages produced significantly lower amounts of mature IL-1β compared to WT controls (WT: 16,113 ± 1441 vs. S1PR1 KO: 10,247 ± 1628 pg/mL) (Figure 4A). To test if the attenuated response was specific to a particulate assembler such as AlOH or resembled a general phenomenon, we used the TL7 agonist IMQ as signal 2 for NLRP3 inflammasome activation, which has previously been described for murine macrophages [41,42]. IMQ alone fail to elicit any response from macrophages. However, similar to the AlOH, we observed an attenuated response in S1PR1 KO macrophages when IMQ was used as an assembler of the NLRP3 inflammasome in combination of LPS (WT: 11,793 ± 2359 vs. S1PR1 KO: 7636 ± 3170 pg/mL) (Figure 4A). This suggested that S1PR1 signaling might regulate NLRP3 inflammasome in a broader sense. We also measured cytokines such as IL-6 (Figure 4B), IL-10 (Figure 4C), and IL-23 (p19/p40) (Figure 4D) as well as the chemokine CCL5 (Figure 4E). Interestingly, S1PR1 KO cells do not differ from WT in any treatment for these factors, except IL-6, which was attenuated in S1PR1 KO macrophages upon LPS stimulation (Figure 4B).

In these experimental conditions, data from S1PR1 KO macrophages suggest that S1PR1 signaling specifically alters NLRP3 inflammasome activation without major effects on other effector functions of macrophages such as cytokine and chemokine production.

### 2.4. Redundant Role of SPHK1 and SPHK2 in NLRP3 Inflammasome Activation

**Figure 5:**
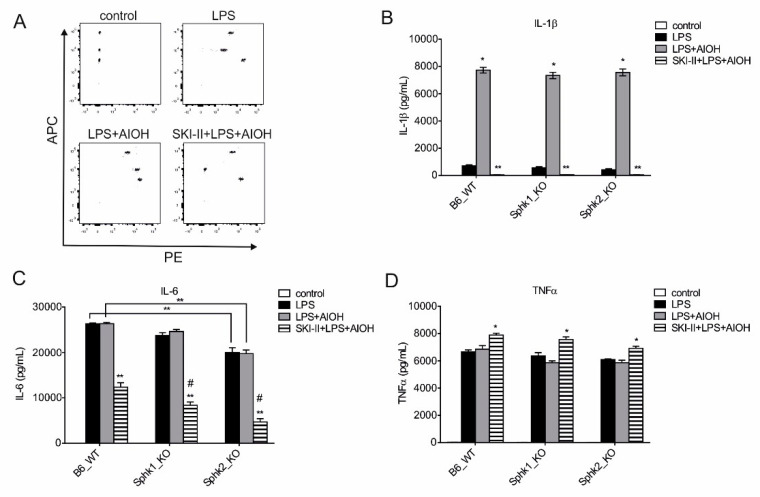
Individual SPHKs are redundant in NLRP3 inflammasome activation. Bone marrow-derived macrophages (BMDMs) from B6 WT, *Sphk1* KO, and *Sphk2* KO were stimulated with LPS alone or in combination with 10 µM SKI-II, 10 µg/mL IMQ, and 100 µg/mL AlOH for 24 h. Cell-free supernatants were harvested and analyzed by CBA. (**A**) Representative FACS dot-plot of phycoerythrin (PE) and allophycocyanine (APC), (**B**) IL-1β, (**C**) IL-6 and (**D**) TNF-α level. Data are means ± SEM, *n* ≥ 4 mice. ^#^, significant to B6 WT; * *p* < 0.05; ** *p* < 0.001; *p* values were calculated using two-way ANOVA with Sidak correction.

Next, we wanted to assign a role to each S1P kinases in NLRP3 inflammasome activation in macrophages. Hence, to dissect the role of SPHK1 and SPHK2 in macrophage inflammasome activation, we compared B6 WT with *Sphk1* KO and *Sphk2* KO macrophages during NLRP3 inflammasome activation. When both signals for NLRP3 inflammasome activation were present, i.e., LPS and AlOH, IL-1β release was again greatly enhanced compared to priming with LPS alone (Figure 5A,B). Genetic deletion of individual S1P kinases showed limited effect on IL-β secretion as both *Sphk1* or *Sphk2* KO macrophages produced comparable amount of IL-1β (Figure 5B). However, mimicking the kinase double KO condition by using the SKI-II inhibitor, NLRP3 inflammasome activity was completely abrogated as only negligible amounts of IL-1β were released from *Sphk1* or *Sphk2* KO macrophages (Figure 5B). We also measured IL-6 release from these macrophages. SPHKs were also required for IL-6 production upon LPS stimulation. Genetic deletion of *Sphk2* or pharmacological blockage of SPHK1 and SPHK2 significantly reduced IL-6 production (Figure 5C). In contrast, TNF-α secretion was largely intact in SPHK KO macrophages, while SKI-II even enhanced TNF-α release in all groups (Figure 5D).

These data highlight the contribution of both SPHK1 and SPHK2 to NLRP3 inflammasome activation and IL-6 production in mouse macrophages.

### 2.5. Clinical Correlation of SPHKs and NLRP3 Inflammasome Expression

**Figure 6:**
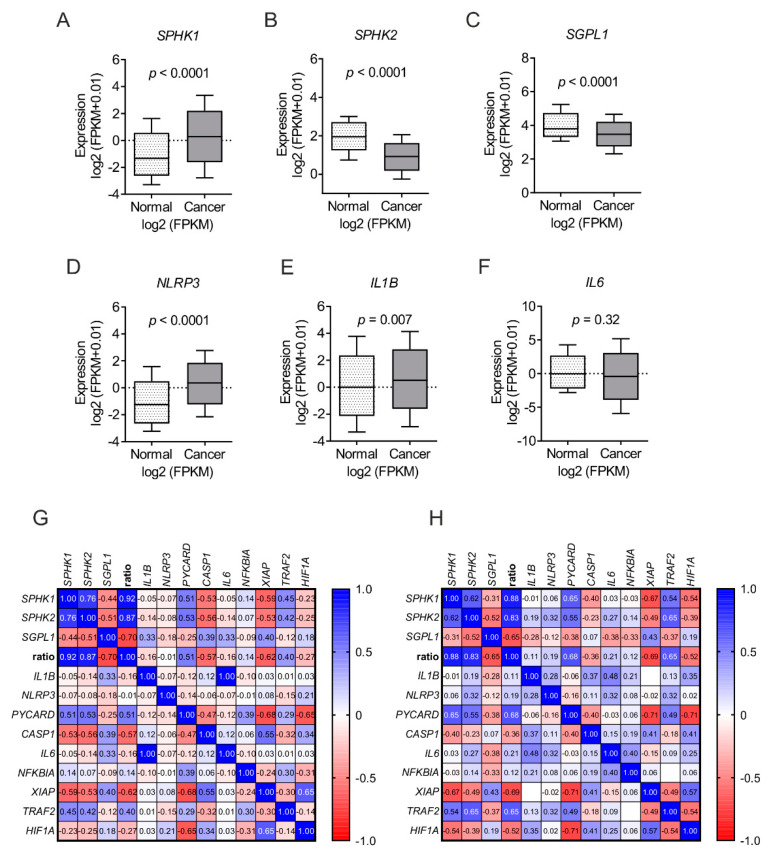
Clinical correlation between SPHKs and NLRP3. The expression of S1P metabolizing enzymes were correlated with that of NLRP3 inflammasome components in kidney renal cell carcinoma and psoriatic skin. Publicly available TCGA-KIRC datasets of cancer and normal adjacent tissue were analyzed for the expression of (**A**) *SPHK1*, (**B**) *SPHK2*, (**C**) *SGPL1*, (**D**) *NLRP3*, (**E**) *IL1B* and (**F**) *IL6* from tumor (cancer; *n* = 535) and normal (normal; *n* = 72) samples. Results are presented as log 2 fragments per kilobase of transcript per million mapped reads (FPKM). Gene expression data in Gene Expression Omnibus (GEO) dataset GSE54456 comprising of (**G**) normal skin (*n* = 82 and (**H**) psoriatic skin (*n* = 92) were analyzed for NLRP3 inflammasome components (*IL1B, NLRP3, PYCARD* and *CASP1*) and NF-κB target genes (*IL6*, *NFKBIA*, *XIAP*, *TRAF2* and *HIF1A*) with an in silico generated S1P production ratio. Spearman *r*-values are indicated. *P* values were calculated using two-tailed Student’s *t*-test.

Finally, to understand the clinical relevance of our finding, we analyzed expression pattern of these genes in an inflammatory condition and a cancer type where both S1P and macrophages have been reported in poor prognosis [43,44,45,46]. We analyzed publicly available datasets of patients comprising 535 tumor samples and 72 control tissue of clear cell renal cell carcinoma [47]. Expression data of genes in cancer and corresponding normal tissues were downloaded from the TCGA-KIRC project [47] via the Genomic Data Commons Data Portal [48] and curated by ENCORI Pan-Cancer Analysis Platform [49]. There was a significantly enhanced expression of *SPHK1* in cancer tissue compared to normal tissue (Figure 6A). The expression of *SPHK2* and *SGPL1* were downregulated in cancer compared to normal tissue (Figure 6B,C). *NLRP3* and *IL1B* were upregulated in cancer tissue compared to normal (Figure 6D,E), while *IL6* expression was unaltered (Figure 6F). Next, we analyzed expression datasets in an inflammatory skin condition, i.e., psoriasis [50,51]. A high throughput sequencing dataset (GSE54456) containing 92 psoriatic and 82 normal skin samples was downloaded from Gene Expression Omnibus (GEO). An in silico S1P production ratio was generated by comparing mean expression of S1P generating, versus S1P degrading enzymes (mean expression (*SPHK1* + *SPHK2*) / mean expression (*SGPL1*) and compared with NLRP3 inflammasome components (*IL1B*, *NLRP3*, *PYCARD,* and *CASP1*) and NF-κB target genes (*IL6*, *NFKBIA*, *XIAP*, *TRAF2,* and *HIF1A*). Apparently a high S1P ratio, which may indicate increased S1P levels, was positively correlated with NLRP3 inflammasome components, except CASP1, which corroborated our findings (Figure 6G,H). In contrast, NF-κB target genes presented a mosaic picture in which *TRAF2* and *NFKBIA* positively correlated and *XIAP* and *HIF1A* negatively correlated with the S1P ratio (Figure 6G,H).

The above datasets suggested a net S1P production due to enhanced expression of cytosolic S1P synthesizing enzyme (*SPHK1*) vs. S1P degrading enzyme (*SGPL1*). This net S1P production was corelated with enhanced expression of NLRP3 inflammasome components (*NLRP3*, *IL1B*). These clinical datasets corroborated our finding of a positive correlation between SPHKs expression and NLRP3 inflammasome components in macrophages.

## 3. Discussion

S1P plays an important role in several inflammatory diseases including inflammation-driven cancers. Therefore, enzymes required for S1P metabolism inadvertently became attractive therapy targets for these diseases. Steady state levels of S1P are controlled not only by S1P biosynthetic enzymes (SPHKs) but also by S1P degradative pathways, such as a S1P-specific lyase (SPGL) and by three lipid phosphatases. The net activity of these enzymes controls the S1P rheostat. In order to understand and assign a pharmacological relevance to S1P metabolic enzymes, we deciphered the role of S1P, upstream of S1PRs, in NLRP3 inflammasome activation and inflammation with the assumption that SPHKs might play an important role in this process. SPHK1 and SPHK2 have overlapping characteristic functions but also differ in their function and localization [52]. The predominant location of SPHK1 is in the cytoplasm, from where it rapidly translocates to the plasma membrane upon stimulation. SPHK1 activity, therefore, leads to “inside-out” S1P signaling in an autocrine and/or paracrine fashion [17,53,54]. S1PR1 can be activated via lateral slide of S1P within the plane of the membrane bilayer to access the receptor binding pocket for activation [55], suggesting that S1P may not be required to be secreted for its autocrine action and S1PR1 blockage. This may explain why W146 may not prevent SPHKs-derived S1P action towards NLRP3 inflammasome (Figure 1A and Figure 2D). However, the release of NF-κB target gene product such as IL-1β and IL-6 was reduced in S1PR1 KO macrophages (Figure 4A,B). This may suggest a role of S1PR1 in NF-κB signaling. Moreover, SPHK1 can also interacts with TRAF2 that causes NF-κB activation [56], which is also a converging point of TLR4 activation by LPS. This supports the idea that SPHK1 may cooperate with TLR4 signaling in inflammatory responses independent of S1PR signaling and S1P production. Furthermore, S1P can also trigger a positive feedback amplification loop via S1PRs activation that increases SPHK1 expression [57], which may also explain increased expression of *NLRP3* and *IL1B* in human macrophages (Figure 2A,B). Noteworthy, it has already been shown that LPS stimulated macrophages produce S1P, which acts as an intracellular secondary messenger rather than an extracellular ligand for S1PRs [58,59]. Furthermore, LPS per se increases NALP3 protein stability without significantly altering steady-state mRNA by degrading its negative regulator F-box protein FBXL2 in human monocytes [60]. SPHK2, on the other hand, can shuttle in and out of the nucleus, due to the presence of nuclear localization and nuclear export signal, and primarily resides at the endoplasmic reticulum [61,62]. S1P generated via SPHK2 can affect cell survival and growth [21,62,63]. Our data provide additional information to SPHK2 and cell survival as *Sphk2* KO macrophages produced reduced amount of IL-6 (Figure 5C), which is also being implicated in cell survival by polarizing cells towards alternatively activated macrophages by HIF-1α activation and upregulation of anti-apoptotic proteins [64,65,66]. We have recently reported that inflammatory stimulated human macrophages show a biphasic response to SPHK2 expression and activity. Of note SPHK2 activity is required for an early inflammatory activity and to dampen late inflammatory responses [67]. We observed that SPHK2 was degraded upon LPS-treatment, which was necessary to allow the full magnitude of early inflammatory cytokine production from human macrophages [67]. These kinetic attributes of SPHK2 might explain the induction of TNF-α, though to a varying degree in human (Figure 1B) and mouse (Figure 5D) macrophages and upon blockage with SKI-II. SPHK2 can also binds to histone H3 in the nucleus and bring about epigenetic regulation of gene expression by nuclear S1P action on histone deacetylases [21]. How and if epigenetic regulations mediated by SPHK2 are also linked to NLRP3 inflammasome activity and inflammation is currently unknown and requires further investigation.

TLR4 activation, or any signal that activates NF-κB, primes NLRP3 inflammasome. Recent studies suggested that LPS not only activates TLR4 signaling but also the hexa-acyl lipid A component of LPS is able to access the cytoplasm, where it activates caspase-11 to signal NLRP3 inflammasome activation independently of TLR4 [68,69]. There are several activators for NLRP3 such as ROS, mitochondrial dysfunction, lysosomal leakage, changes in cellular calcium flux, membrane pore formation, and potassium efflux. Potassium release is found in association with all NLRP3 activators and apparently a culture medium low in potassium is sufficient to trigger NLRP3 activation. [35]. Imiquimod, a TLR7 agonist, can induce the NLRP3 inflammasome independent of potassium using NEK7 as a ROS sensor [41]. Intriguingly, IMQ enhances the anti-inflammatory potential of mouse macrophages by potentiating IL-10 release (Figure 4C). Unlike in mouse cells, IMQ does not act as a signal 2 for NLRP3 inflammasome activation in primary human macrophages. These differences might be due to intrinsic variances between human and mouse NLRP3 inflammasome activation [70] or might be due to cooperation of S1PRs signaling with TLR4 and TLR7, which is a contention of further investigation.

There is redundancy in the action of SPHK1 and SPKH2 in terms of NLRP3 inflammasome activation as activity of either of these enzymes restored NLRP3 activity (Figure 5B). This redundancy could also be due to various factors including expression regulation of individual NLRP3 components since *Sphk1* deficiency attenuate *Il1b* expression (Figure 3A), whereas NLRP3 activity was unaltered (Figure 3B and Figure 5B). However, the functional redundancy is not new for these kinases as it has been reported in the literature that mice lacking either *Sphk1* or *Sphk2* are viable, fertile and have no obvious abnormality [71,72]. In contrast double knockout show prenatal death, owing to severe defects in neurogenesis and angiogenesis [72]. Furthermore, compensatory upregulation of *Sphk1* expression was observed in *Sphk2* deficient macrophages [73].

SPHKs not only catalyze similar enzymatic reaction to produce anti-apoptotic S1P, while consuming pro-apoptotic ceramide, they show interdependence on each other in S1P signaling even in pharmacological settings. For example fingolimod (FTY720), one of the widely used pharmacological compounds for antagonizing S1PR1, is processed by SPHK2 to fingolimod-phosphate, which serves as a potent agonist of S1PR1 [74,75] as well as an inhibitor of SPHK1 [76]. Since most S1P actions are mediated by S1PRs, currently, the major class of drugs interfering with S1P signaling are directed towards S1PRs rather than metabolic enzymes involved in S1P biosynthesis/processing. There is no question that targeting a specific S1PR would provide selectivity with some off-target effects. Pharmacologically, targeting enzymes is more potent due to high specificity and does not pose a threat of target activation, which is an issue when targeting G protein-coupled receptors such as S1PRs. For example, fingolimod has been shown to be a potent agonist of S1PR1. However, on lymphocytes, fingolimod can downregulate S1PR1, thus preventing the egress of these cells from lymphoid tissues. This dichotomy (agonism/antagonism) of fingolimod on S1PR1 reduces the infiltration of lymphocytes into the central nervous system, thereby blocking their undesirable effects [74,77,78]. Evidently, there are several options in terms of the nature of enzyme inhibitor such as competitive, non-competitive, uncompetitive and irreversible inhibitors that can be designed and used for optimal efficacy and in a specific scenario. The present study, describing the useful contribution of SPHKs in inflammasome assembly/activation, is a primer in this direction.

Taking an extra stride towards translation research, we used both human and mouse cells. We exploited both genetic deletion and pharmacological blockade of SPHK1 and SPHK2. One of the reasons of using genetically deleted SPHK1 and SPHK2 macrophages, in combination/addition to pharmacological inhibitors, was to avoid side effects reported for pharmacological inhibitors. Conversely, the genetic deletion of *Sphk2* showed minor effects on disease progression in the mouse model of TNF-α-induced arthritis, whereas the pharmacologic inhibition with the SPHK2 inhibitor ABC294640 augmented disease severity [79]. Nevertheless, while SKI-II exhibits no inhibitory action on other kinases including PI3K, PKCα and ERK2 [27,80,81,82], side effects of SKI-II cannot be ruled out since *Sphk1* and *Sphk2* double KO macrophages have been shown to have redundant pro-inflammatory responses to LPS [73]. Furthermore, our experimental conditions do not rule out effects of upstream metabolites i.e., ceramide accumulation due to attenuated SPHK activities. It was shown that a lipotoxicity-associated increase of ceramide provokes caspase-1 cleavage and NLRP3 inflammasome activation in macrophages and adipose tissue [83], suggesting that the NLRP3 inflammasome can sense elevated intracellular ceramide. Furthermore, age-related increased thymic ceramides also support NLRP3 inflammasome-dependent caspase-1 activation [84]. Nevertheless, these studies also serendipitously support our findings and underscore the role of SPHKs in NLRP3 inflammasome activation.

Monocytes and macrophages are critical players psoriasiform skin inflammation [43,85] and in the tumor microenvironment [44], and they are being implicated to play tumor-promoting roles in human renal cell carcinoma (RCC) patients. Expectedly, *IL1B* expression was enhanced in blood monocytes isolated from RCC patients [86] and in tumor tissue (Figure 6E). *SGPL1* expression was also downregulated in RCC tumor samples (Figure 6C), which supports our narrative. However, the precise role of this enzyme in cancer has not been established yet as its expression was reduced in intestinal cancer [87] and metastatic tumors [88], whereas increased in ovarian cancer [89] and in chemotherapeutic resistant ovarian tumors [90]. *SPHK2* seem to be downregulated in RCC tumors (Figure 6B), which goes in line with studies suggesting that although both SPHK1 and SPHK2 use the same physiologic substrate and generate S1P, SPHK2 might have a role opposite to that of SPHK1 as over-expression of *SPHK2* suppresses cell growth and enhances apoptosis [61,91]. Furthermore, the expression profile of *SPHK2* and *IL6* in human RCC tumors correlated with the finding of mouse macrophages (Figure 5C), suggesting that the role of this kinase in IL-6 production has pathophysiological relevance. SPHK1 overexpression contributes to sunitinib resistance in clear cell renal cell carcinoma [92]. In spite of a positive correlation between the S1P metabolic enzymes and inflammation in RCC, globally targeting S1P using the anti-S1P monoclonal antibody sonepcizumab failed in a phase II clinical trial of metastatic renal cell carcinoma [45]. On the other hand, hispidulin, a polyphenolic flavonoid, suppressed tumor growth and metastasis of RCC cells by interfering with the phosphorylation and translocation of SPHK1, thereby inhibiting its activity without affecting mRNA or protein expression [93].

Being an inflammatory dermatosis, psoriasis shows hallmark feature of inflammation including elevated levels of active, phosphorylated NF-κB. NF-κB has been hypothesized to connect the altered keratinocyte and immune cell behavior that characterizes the psoriatic milieu. Indeed, recent evidence suggests that the activation of particular NF-κB target genes is highly complex and dependent on selective gene regulation in distinct pathological settings [94]. Similarly, we noted that in psoriatic skin though there was a positive correlation with SPHKs and NLRP3 inflammasome components, some of the them are targets of NF-κB signaling. Other NF-κB target genes showed mosaic picture, both in healthy and psoriatic skin, indicating that regulation of NLRP3 inflammasome components might involve other signaling pathways than NF-κB alone (Figure 6G,H). Indeed, the exact molecular mechanisms how SPHKs affect NF-κB signaling in macrophage is still not fully understood and may require further detailed investigation.

In conclusion, we provide compelling, new evidence for a direct involvement of SPHKs in macrophage NLRP3 inflammasome activation and provide a rationale for therapeutic targeting these kinases in cancer and inflammation.

## 4. Materials and Methods

### 4.1. Mice and Reagents

*S1pr1* KO (*S1pr1^fl/fl^Lyz2^Cre/Cre^*) and corresponding WT (*S1pr1^fl/fl^Lyz2^Cre/wt^*) on C57BL/6 background have been described before [46]. *Sphk1* KO [95,96] and *Sphk2* KO [12,97] from Novartis were described before and were backcrossed for at least 10 generations into a C57BL/6 background. Mice were kept under standard pathogen-free conditions with ad libitum food (regular chow diet) and water, and a 12:12 h light:dark cycle. Organ removal and animal care were performed in accordance with the EU Directive 86/609 EEC and German Protection of Animals Act. No additional animal ethics approval is needed for organ removal for subsequent isolation of primary murine cells. Ultrapure lipopolysaccharide (LPS) from *E. coli* 0111:B4 was purchased from InvivoGen (San Diego, CA, USA), SKI-II (4-[[4-(4-chlorophenyl)-2-thiazolyl]amino]-phenol), CAY 10621 (2,2-dimethyl-4S-(1-oxo-2-hexadecyn-1-yl)-1,1-dimethylethyl ester-3-oxazolidinecarboxylic acid), VPC 23019 ((R)-2-amino-3-((3-octylphenyl)amino)-3-oxopropyl dihydrogen phosphate) were bought from Cayman Chemical (Ann Arbor, MI, USA), W146 (R-3-amino-4-(3-hexylphenylamino)-4-oxobutylphosphonic acid hydrate) and DMSO was purchased from Sigma-Aldrich (München, Germany). The source of all other reagents was mentioned at their respective use.

### 4.2. RNA isolation, Reverse Transcription, and Quantitative Real-Time PCR

Macrophages were snap frozen in liquid nitrogen or lysed directly in PeqGold^®^ (Peqlab Biotechnology, Erlangen, Germany). Isolation of RNA from cells was performed according to the manufacturer’s instructions and quantified using the NanoDrop spectrophotometer (NanoDrop, Wilmington, DE, USA). RNA was transcribed into cDNA for mRNA analysis using the Fermentas reverse transcriptase kit (ThermoFisher Scientific, Karlsruhe, Germany) according to the manufacturer’s instructions. Real-time quantitative PCR (qPCR) was performed using the SYBR green on CFX96™ Real-Time PCR Detection System (Bio-Rad Laboratories, Munich, Germany) and the QuantStudio 5 Real-Time PCR System (Applied Biosystems, Damstadt, Germany). QuantiTect primer assays (QIAGEN, Hilden, Germany) were used to detect human *NLRP3*, *PYCARD*, *B2M*. All other primers were from Biomers GmbH, Germany. Mouse *Il1b* 5′-TGAAATGCCACCTTTTGACA-3′ and 5′-AGCTTCTCCACAGCCACAAT-3′, mouse *Il18* 5′-GGCTGCCATGTCAGAAGACT-3′ and 5′-GTGAAGTCGGCCAAAGTTGT-3′, mouse *Nlrp3* 5′-ATTGCTGTGTGTGGGACTGA-3′ and 5′-AACCAATGCGAGATCCTGAC-3′, mouse *Pycard* 5′-ACATGGGCTTACAGGAGCTG-3′ and 5′-GCTGGTCCACAAAGTGTCCT-3′, mouse *Casp1* 5′-GCTTGAAAGACAAGCCCAAG-3′ and 5′-GGCCTTCTTAATGCCATCAT-3′, mouse *Rsp27a* 5′-GACCCTTACGGGGAAAACCAT-‘3 and 5′-AGACAAAGTCCGGCCATCTTC-3′, human *IL1B* 5′-TTCGACACATGGGATAACGAGG-3′ and 5′-TTTTTGCTGTGAGTCCCGGAG-3′ and human *TNFA* 5′-GAGGCCAAGCCCTGGTATG-3′ and 5′-CGGGCCGATTGATCTCAGC-3′. Relative mRNA expression was calculated using either the CFX-Manager^TM^ v3.2 software (Bio-Rad Laboratories) or the QuantStudio^TM^ Design and Analysis software v1.5 (Applied Biosystems) using the ΔΔC_t_ method and normalized to either *B2M* (human) or *Rps27a* (mouse) as housekeeping genes.

### 4.3. Cytokine Measurements

To determine cytokine levels in cell culture supernatants human IL-1β, and TNF-α or murine IL-1β, IL-, IL-10, IL-23 (p19/p40), TNF-α and CCL5 Cytometric Bead Array Flex Sets (BD Biosciences, Franklin Lakes, NJ, USA) were used. Samples were acquired with a LSRII/Fortessa flow cytometer (BD Biosciences) and data were analyzed using BD Biosciences FCAP software (V3.0).

### 4.4. Macrophage Culturing and Stimulation

Primary human monocytes (PBMCs) were isolated from buffy coats of anonymous healthy donors obtained from DRK Blutspendedienst (Baden-Würtemberg-Hessen, Frankfurt, Germany) using Ficoll-Hypaque gradients (PAA Laboratories, Cölbe, Hessen, Germany). Cells were washed twice with PBS and plated onto high-adherence culture dishes (Sarstedt, Nümbrecht, Germany). After culturing PBMCs for 1 h in RPMI 1640 media containing with 100 U/mL penicillin and 100 µg/mL streptomycin, non-adherent cells were washed away, and remaining monocytes were cultured in media containing 5% AB-positive human serum for 7 days to allow the differentiation towards macrophages.

Bone marrow was isolated from tibia and femur of different mice strains described in the text and 4 × 10^6^ bone marrow cells were plated in a 6-well plate in each well and were incubated in RPMI 1640 containing 10% FCS, 100 U/mL penicillin, 100 µg/mL streptomycin. For differentiation into macrophages, cells were cultured in the presence of 20 ng/mL M-CSF and 20 ng/mL GM-CSF (ImmunoTools, Friesoythe, Germany) for 7 days. Medium was replaced every second days and differentiated bone marrow-derived macrophages were stimulated with 10–100 ng/mL of LPS in combination with various stimuli described in figure legends for various time points.

### 4.5. Analysis of Publicly Available Datasets of Gene Expression in Renal Cell Carcinoma and Psoriasis

Publicly available datasets comprising 535 tumor samples and 72 control tissues of clear cell renal cell carcinoma patients [47] were obtained from TCGA-KIRC project [47] via Genomic Data Commons Data Portal [48] and analyzed by ENCORI Pan-Cancer Analysis Platform. Gene Expression Omnibus (GEO) datasets comprising 92 psoriatic and 82 normal skin samples (GSE54456) were downloaded and analyzed either via GEO2R [98] or GDSBrowser [99]. Expression profiles of S1P metabolizing enzymes along with NLRP3 inflammasome components and NF-κB target genes were further analyzed and plotted using GraphPad Prism v8.

### 4.6. Statistical analysis

All data are presented as mean values ± SEM of at least two independent experiments. Statistical analyses were performed in GraphPad Prism *v*. 6 using one-sample *t*-test, two-tailed Student’s *t*-test, two-way ANOVA with Sidak correction with two-tailed *p* values as indicated in the figure legends. Asterisks indicate significant differences between different groups (* *p* < 0.05, ** *p* < 0.01).

## Abbreviations

NLRP3	NACHT, LRR and PYD domains-containing Protein 3
PYCARD	PYD And CARD Domain-Containing Protein
S1P	Sphingosine-1-phosphate
SPHK	Sphingosine Kinase
SGPL	S1P lyase
AlOH	Aluminum Hydroxide
WT	Wild type
KO	Knock out
IL	Interleukin
TNF	Tumor-necrosis factor
RCC	Renal cell carcinoma
